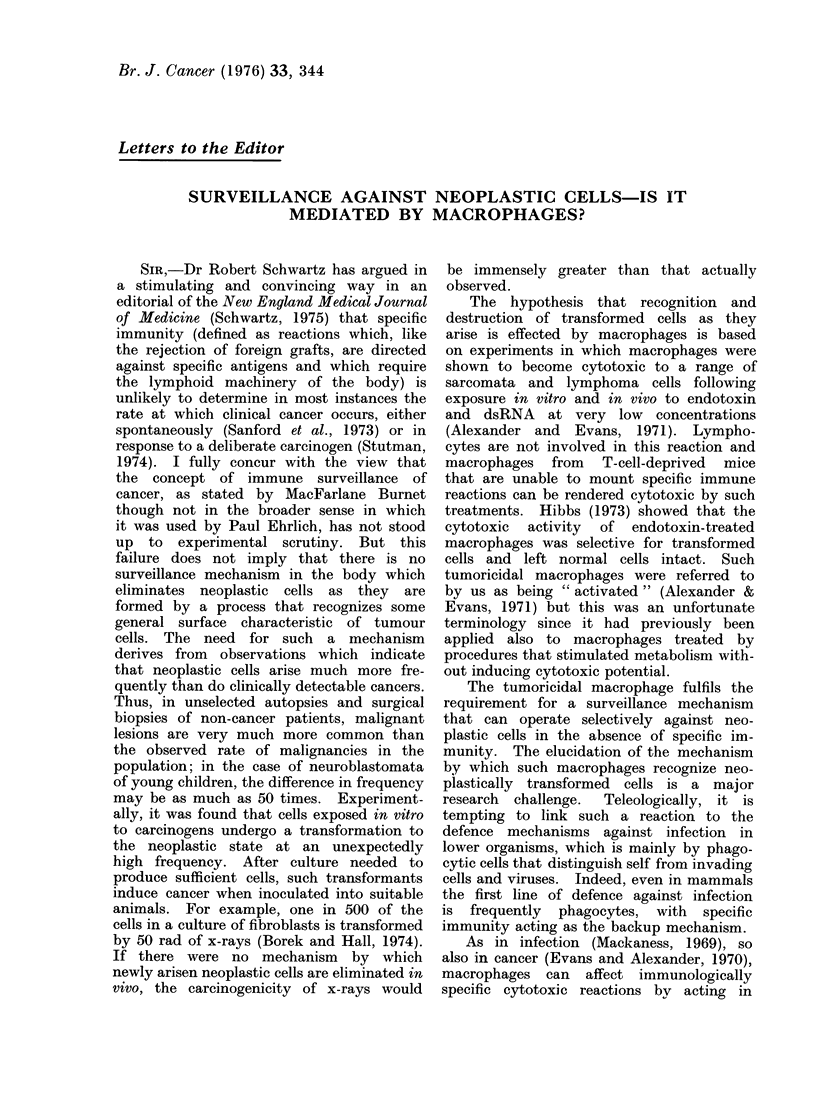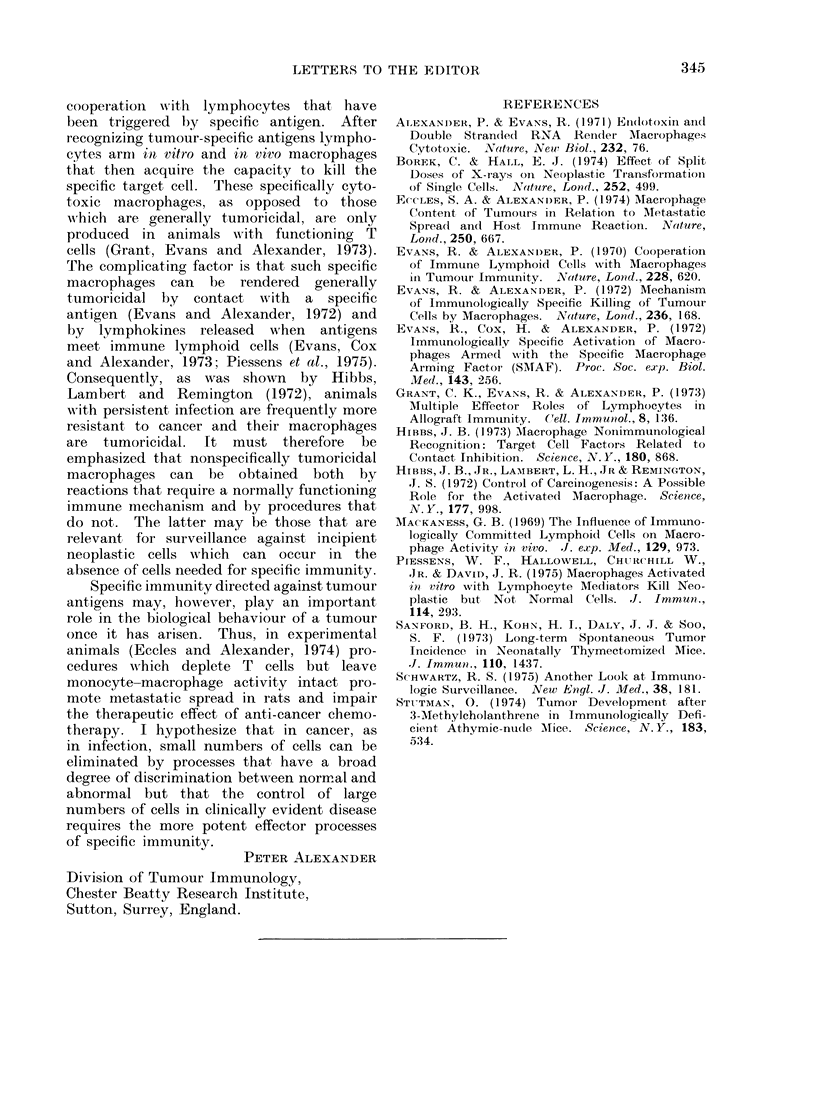# Letter: Surveillance against neoplastic cells-is it mediated by macrophages?

**DOI:** 10.1038/bjc.1976.50

**Published:** 1976-03

**Authors:** P. Alexander


*Br. J. Cancer* (1976) **33**, 344

### *Letters to the Editor*

### SURVEILLANCE AGAINST NEOPLASTIC CELLS—IS IT MEDIATED BY MACROPHAGES?

SIR,—Dr Robert Schwartz has argued in a stimulating and convincing way in an editorial of the *New England Medical Journal of Medicine* (Schwartz, 1975) that specific immunity (defined as reactions which, like the rejection of foreign grafts, are directed against specific antigens and which require the lymphoid machinery of the body) is unlikely to determine in most instances the rate at which clinical cancer occurs, either spontaneously (Sanford *et al.*, 1973) or in response to a deliberate carcinogen (Stutman, 1974). I fully concur with the view that the concept of immune surveillance of cancer, as stated by MacFarlane Burnet though not in the broader sense in which it was used by Paul Ehrlich, has not stood up to experimental scrutiny. But this failure does not imply that there is no surveillance mechanism in the body which eliminates neoplastic cells as they are formed by a process that recognizes some general surface characteristic of tumour cells. The need for such a mechanism derives from observations which indicate that neoplastic cells arise much more frequently than do clinically detectable cancers. Thus, in unselected autopsies and surgical biopsies of non-cancer patients, malignant lesions are very much more common than the observed rate of malignancies in the population; in the case of neuroblastomata of young children, the difference in frequency may be as much as 50 times. Experimentally, it was found that cells exposed *in vitro* to carcinogens undergo a transformation to the neoplastic state at an unexpectedly high frequency. After culture needed to produce sufficient cells, such transformants induce cancer when inoculated into suitable animals. For example, one in 500 of the cells in a culture of fibroblasts is transformed by 50 rad of x-rays (Borek and Hall, 1974). If there were no mechanism by which newly arisen neoplastic cells are eliminated *in vivo*, the carcinogenicity of x-rays would be immensely greater than that actually observed.

The hypothesis that recognition and destruction of transformed cells as they arise is effected by macrophages is based on experiments in which macrophages were shown to become cytotoxic to a range of sarcomata and lymphoma cells following exposure *in vitro* and *in vivo* to endotoxin and dsRNA at very low concentrations (Alexander and Evans, 1971). Lymphocytes are not involved in this reaction and macrophages from T-cell-deprived mice that are unable to mount specific immune reactions can be rendered cytotoxic by such treatments. Hibbs (1973) showed that the cytotoxic activity of endotoxin-treated macrophages was selective for transformed cells and left normal cells intact. Such tumoricidal macrophages were referred to by us as being " activated " (Alexander & Evans, 1971) but this was an unfortunate terminology since it had previously been applied also to macrophages treated by procedures that stimulated metabolism without inducing cytotoxic potential.

The tumoricidal macrophage fulfils the requirement for a surveillance mechanism that can operate selectively against neoplastic cells in the absence of specific immunity. The elucidation of the mechanism by which such macrophages recognize neoplastically transformed cells is a major research challenge. Teleologically, it is tempting to link such a reaction to the defence mechanisms against infection in lower organisms, which is mainly by phagocytic cells that distinguish self from invading cells and viruses. Indeed, even in mammals the first line of defence against infection is frequently phagocytes, with specific immunity acting as the backup mechanism.

As in infection (Mackaness, 1969), so also in cancer (Evans and Alexander, 1970), macrophages can affect immunologically specific cytotoxic reactions by acting in cooperation with lymphocytes that have been triggered by specific antigen. After recognizing tumour-specific antigens lymphocytes arm *in vitro* and *in vivo* macrophages that then acquire the capacity to kill the specific target cell. These specifically cytotoxic macrophages, as opposed to those which are generally tumoricidal, are only produced in animals with functioning T cells (Grant, Evans and Alexander, 1973). The complicating factor is that such specific macrophages can be rendered generally tumoricidal by contact with a specific antigen (Evans and Alexander, 1972) and by lymphokines released when antigens meet immune lymphoid cells (Evans, Cox and Alexander, 1973; Piessens *et al.*, 1975). Consequently, as was shown by Hibbs, Lambert and Remington (1972), animals with persistent infection are frequently more resistant to cancer and their macrophages are tumoricidal. It must therefore be emphasized that nonspecifically tumoricidal macrophages can be obtained both by reactions that require a normally functioning immune mechanism and by procedures that do not. The latter may be those that are relevant for surveillance against incipient neoplastic cells which can occur in the absence of cells needed for specific immunity.

Specific immunity directed against tumour antigens may, however, play an important role in the biological behaviour of a tumour once it has arisen. Thus, in experimental animals (Eccles and Alexander, 1974) procedures which deplete T cells but leave monocyte–macrophage activity intact promote metastatic spread in rats and impair the therapeutic effect of anti-cancer chemotherapy. I hypothesize that in cancer, as in infection, small numbers of cells can be eliminated by processes that have a broad degree of discrimination between normal and abnormal but that the control of large numbers of cells in clinically evident disease requires the more potent effector processes of specific immunity.

PETER ALEXANDER

Division of Tumour Immunology,
Chester Beatty Research Institute,
Sutton, Surrey, England.

REFERENCES

ALEXANDER, P. & EVANS, R. (1971) Endotoxin and Double Stranded RNA Render Macrophages Cytotoxic. *Nature, New Biol.*, **232**, 76.

BOREK, C. & HALL, E. J. (1974) Effect of Split Doses of X-rays on Neoplastic Transformation of Single Cells. *Nature, Lond.*, **252**, 499.

ECCLES, S. A. & ALEXANDER, P. (1974) Macrophage Content of Tumours in Relation to Metastatic Spread and Host Immune Reaction. *Nature, Lond.*, **250**, 667.

EVANS, R. & ALEXANDER, P. (1970) Cooperation of Immune Lymphoid Cells with Macrophages in Tumour Immunity. *Nature, Lond.*, **228**, 620.

EVANS, R. & ALEXANDER, P. (1972) Mechanism of Immunologically Specific Killing of Tumour Cells by Macrophages. *Nature, Lond.*, **236**, 168.

EVANS, R., COX, H. & ALEXANDER, P. (1972) Immunologically Specific Activation of Macrophages Armed with the Specific Macrophage Arming Factor (SMAF). *Proc. Soc. exp. Biol. Med.*, **143**, 256.

GRANT, C. K., EVANS, R. & ALEXANDER, P. (1973) Multiple Effector Roles of Lymphocytes in Allograft Immunity. *Cell. Immunol.*, **8**, 136.

HIBBS, J. B. (1973) Macrophage Nonimmunological Recognition: Target Cell Factors Related to Contact Inhibition. *Science, N.Y.*, **180**, 868.

HIBBS, J. B., JR., LAMBERT, L. H., JR & REMINGTON, J. S. (1972) Control of Carcinogenesis: A Possible Role for the Activated Macrophage. *Science, N.Y.*, **177**, 998.

MACKANESS, G. B. (1969) The Influence of Immunologically Committed Lymphoid Cells on Macrophage Activity *in vivo*. *J. exp. Med.*, **129**, 973.

PIESSENS, W. F., HALLOWELL, CHURCHILL W., JR. & DAVID, J. R. (1975) Macrophages Activated *in vitro* with Lymphocyte Mediators Kill Neoplastic but Not Normal Cells. *J. Immun.*, **114**, 293.

SANFORD, B. H., KOHN, H. I., DALY, J. J. & SOO, S. F. (1973) Long-term Spontaneous Tumor Incidence in Neonatally Thymectomized Mice. *J. Immun.*, **110**, 1437.

SCHWARTZ, R. S. (1975) Another Look at Immunologic Surveillance. *New Engl. J. Med.*, **38**, 181.

STUTMAN, O. (1974) Tumor Development after 3-Methylcholanthrene in Immunologically Deficient Athymic-nude Mice. *Science, N.Y.*, **183**, 534.